# Supplementary material for: BRSET: A Brazilian Multilabel Ophthalmological Dataset of Retina Fundus Photos
Source: PLOS Digit Health. 2024 Jul 11;3(7):e0000454. doi: 10.1371/journal.pdig.0000454 (PMC11239107; doi:10.1371/journal.pdig.0000454)
Supplement: S1 File — (DOCX) [file pdig.0000454.s001.docx]

Supporting information

Data Dictionary – description of BRSET columns

| **Group** | **Column Name** | **Description** |
| --- | --- | --- |
| Descriptive and Demographic fields | *image_id* | Image identifier. |
|  | *patient_id* | Patient identifier |
|  | *camera* | Retinal camera (Canon CR or NIKON NF5050). |
|  | *patient_age* | Age of patient in years. |
|  | *comorbidities* | Free text of self-referred clinical antecedents. |
|  | *diabetes_time* | Self-referred time of diabetes diagnosis in years. |
|  | *insulin_use* | Self-referred use of insulin (yes or no). |
|  | *patient_sex* | Enumerated values: 1 for male and 2 for female. |
|  | *exam_eye* | Enumerated values: 1 for the right eye and 2 for the left eye. |
|  | *nationality* | Patient's nationality. |
| Anatomical parameters | *optic_disc* | Enumerated values: 1 for normal and 2 for abnormal. |
|  | *vessels* | Enumerated values: 1 for normal and 2 for abnormal. |
|  | *macula* | Enumerated values: 1 for normal and 2 for abnormal. |
| Diabetes | *diabetes* | Diabetes diagnosis |
| Diabetic retinopathy classification | *DR_ICDR* | International Clinic Diabetic Retinopathy classification with enumerated values from 0 to 4:   - 0 No retinopathy. - 1 Mild non-proliferative diabetic retinopathy. - 2 Moderate non-proliferative diabetic retinopathy. - 3 Severe non-proliferative diabetic retinopathy. - 4 Proliferative diabetic retinopathy and post-laser status. |
|  | *DR_SDRG* | Scottish Diabetic Retinopathy Grading Scheme classification with enumerated values from 0 to 4:   - 0 No retinopathy. - 1 Mild Background. - 2 Moderate Background. - 3 Severe non-proliferative or pre-proliferative diabetic retinopathy. - 4 Proliferative diabetic retinopathy and post-laser status. |
| Quality parameters | *focus* | Enumerated values: 1 for normal and 2 for abnormal. |
|  | *illumination* | Enumerated values: 1 for normal and 2 for abnormal. |
|  | *image_field* | Enumerated values: 1 for normal and 2 for abnormal. |
|  | *artifacts* | Enumerated values: 1 for normal and 2 for abnormal. |
| Classification parameters | *diabetic_retinopathy* | 1 present and 0 absent. |
|  | *macular_edema* | 1 present and 0 absent. |
|  | *scar* | 1 present and 0 absent. |
|  | *nevus* | 1 present and 0 absent. |
|  | *amd* | 1 present and 0 absent. |
|  | *vascular_occlusion* | 1 present and 0 absent. |
|  | *hypertensive_retinopathy* | 1 present and 0 absent. |
|  | *drusens* | 1 present and 0 absent. |
|  | *hemorrhage* | 1 present and 0 absent. |
|  | *retinal_detachment* | 1 present and 0 absent. |
|  | *myopic_fundus* | 1 present and 0 absent. |
|  | *increased_cup_disc* | 1 present and 0 absent. |
|  | *other* | 1 present and 0 absent. |
