# Supplementary material for: BRSET: A Brazilian Multilabel Ophthalmological Dataset of Retina Fundus Photos
Source: PLOS Digit Health. 2024 Jul 11;3(7):e0000454. doi: 10.1371/journal.pdig.0000454 (PMC11239107; doi:10.1371/journal.pdig.0000454)
Supplement: Attachment — Submitted filename: Rebuttal Letter.docx [file pdig.0000454.s002.docx]

Dear Editor,

Thank you for handling our article and for providing thorough and thoughtful comments that have helped us improve our work. We have revised the manuscript based on the reviewer's suggestions and provided a point-by-point response below. Therein, the Reviewers’ comments are in bold, and our responses are in italics.

To ease the review process, we append a revised version of the manuscript, in which changes are highlighted in red. We hope that you will find the revised version of our work suitable for publication after this round of review.

**Journal Requirements:**

**1. Please provide separate figure files in .tif or .eps format only and remove any figures embedded in your manuscript file. Please also ensure all files are under our size limit of 10MB.**

*We reviewed the journal requirements removed the figures embedded in the manuscript, and submitted them in TIF format.*

**Reviewer #1: Nakayama et. al. published BRSET, an multilevel ophthalmological dataset containing data from the unrepresented Brazil and Latin America population. They performed an exploratory data analysis on BRSET and propose the use of machine learning algorithms for disease and sex classification.**

**The data curation and availability of the BRSET dataset is of high importance. However the authors failed to demonstrate the novelty in their approach or draw conclusions from the results driven. The data analysis presented is superficial. The results presented by ML algorithms are average and almost random - F1 and AUC close to 0.6 is not much better than by chance.**

*Thank you for your valuable feedback and the opportunity to clarify the advancements and contributions of our research. We acknowledge your concerns regarding the novelty of the initial approach and the depth of analysis in our study. In response, we have undertaken significant revisions in our methodology, model selection, and analysis to address these issues comprehensively:*

*1. Enhanced Methodological Approach:*

*In our revised version, we have transitioned from the foundational Dino V2 model to ConvNext V2 (Based in Convolutional Neural Networks) architecture for analyzing the BRSET dataset. This shift as stated in the paper, is not merely a change in the computational model but represents a methodological advancement in handling retina fundus photo analysis. ConvNext V2 introduces several innovations over its predecessors, including layer normalization and expanded kernel sizes, which significantly improve feature extraction and model scalability. This choice was motivated by the need to surpass the baseline performances from previous studies and to harness the latest advancements in deep learning for medical imaging.*

*2. In-Depth Data Analysis:*

*We have expanded our data analysis to include a wider range of classification tasks, encompassing binary and three-class diabetic retinopathy diagnosis, diabetes mellitus prediction, and sex prediction. This broader scope allows for a more comprehensive evaluation of the dataset's utility and the model's capability in extracting clinically relevant features from retina images.*

*3. Improved Model Performance:*

*The results obtained with ConvNext V2, as detailed in the new version of the manuscript, significantly exceed the performance benchmarks set by the initial approach and other models from previous works using our dataset. For instance, in binary diabetic retinopathy diagnosis, ConvNext V2 achieved an AUC of 0.97 and a Macro F1-score of 0.89, demonstrating a substantial improvement over the previous metrics. These enhancements are not limited to a single task but are consistent across all classification objectives, underscoring the model's robustness and the efficacy of our revised analytical strategy.*

*4. Novel Contributions and Insights:*

*Beyond performance metrics, our study now provides novel insights into model interpretability through the use of saliency maps. These visualizations offer a deeper understanding of the model's decision-making processes, highlighting the specific image features that influence diagnostic predictions. This aspect of our research not only advances the field of medical imaging analysis but also contributes to the broader discourse on AI transparency and interpretability in clinical applications.*

**The method descriptions need to be substantially developed. The choice of the feature extraction approach using Dino V2 needs to be addressed. The authors did not explain how they went from an input matrix image to a feature vector with size 768 using Dino V2.**

*Since the results of the deep learning approach using Convnext v2 improved significantly the use of embeddings from Dino V2, we decided to drop from the paper the results of DinoV2, and the whole approach to focus on the deep learning results using Convnext V2.*

**In fact, what are the initial image sizes? Are all images the same size? Was any pre-processing employed? Data description and preprocessing needs to be addressed.**

*Thank you for your insightful inquiries concerning the data description and preprocessing techniques employed in our study. Your questions highlight essential aspects of our research methodology, and we appreciate the opportunity to provide further clarity.*

*In the revised version of our study, we have meticulously addressed the data preprocessing steps to ensure a comprehensive understanding. As explained in the new incorporated section called “Data Preprocessing”, the BRSET dataset comprises images with varying dimensions, with heights ranging from 874 to 2304 pixels and widths from 951 to 2984 pixels. Recognizing the importance of uniform input sizes for deep learning models, we standardized the image dimensions by first resizing all images to 226x226 pixels. This resizing was followed by a random cropping process to achieve a consistent input size of 224x224 pixels for each image. Furthermore, to normalize the pixel values and facilitate model training, we scaled the pixel values to a range between 0 and 1 by dividing each pixel value by 225.*

**The authors mention that they are the only ophthalmological dataset from Latin America and Brazil, however they present in Table 1 a brazilian dataset named DR1/DR2. The authors should say how BRSET is different from this data. There is no reference for DR1/DR2 dataset, or any other dataset in this table.**

*We agree with the reviewer for the comment and added a paragraph to elucidate the fact that the BRSET is not the first but the dataset pioneer in multi-diagnosis and demographic data.*

*The BRSET stands as a pioneering initiative within Latin America's ophthalmological landscape, offering a dataset that distinguishes itself by its multifaceted approach. While previous datasets have existed within the region, BRSET elevates the standard by encompassing a diverse spectrum of diagnoses and sociodemographic categories. In essence, it marks a significant advancement as the inaugural repository to comprehensively integrate multiple diagnoses alongside sociodemographic parameters.*

*We also added the dataset references in the table.*

**The authors commented on the quality of the images, where around 12.2% presented some quality problem. However, they do not inform what was done with these images. Were they used when training the ML algorithms? How were the quality issues addressed?**

*In terms of data quality, we acknowledge that maybe excluding the bad quality images for this analysis may lead to better results; however, we think that those results may not reflect how a model would perform in a real set in some settings, where maybe some images may not have an optimal quality. We clarified that we are using the full set of images in the section Data Preprocessing, where we say “The BRSET dataset was stratified based on labels and divided into training (70%), validation (within training set: 20%), and testing sets (30%)* ***using all the images available in the dataset****.”*

**Authors used SVC and LR to classify diseases and patient sex. However, there are several deep learning approaches that looks more suitable and showed better performance such as the ones presented in references [1, 2]. Why did the authors decided to employ these techniques that underperformed when compared with the literature?**

*Thank you for your constructive feedback regarding our choice of models in the initial version of our study. We initially employed Support Vector Machines (SVM) and Logistic Regression (LR) to establish baseline performance metrics for the BRSET dataset. This approach was aimed at providing a straightforward, lightweight benchmark for future research, acknowledging the accessibility and computational efficiency of these models.*

*However, we recognized the limitations of SVM and LR in capturing the complex patterns inherent in medical imaging data, as evidenced by their relatively underwhelming performance compared to more sophisticated methodologies referenced in the literature. This realization led us to pivot towards employing advanced deep learning techniques, specifically ConvNext V2, in our revised approach.*

*The shift to ConvNext V2 was motivated by its proven capability in handling high-dimensional data and its architectural advancements over traditional models. This choice aligns with the evolving landscape of medical image analysis, where deep learning models have consistently demonstrated superior performance in extracting nuanced features critical for accurate disease classification and patient characterization.*

*Our updated results, employing ConvNext V2, present a substantial improvement over the baseline models and align with the high standards of performance documented in the previous version of the manuscript.*

**Readability suggestions:**

**- Table 4 does not seem necessary and the available features can be easily described in the text or in a supplement material.**

*We moved Table 4 to the supplemental file.*

**- Simple average and std dev for age feature can summarize Fig 1.**

*We removed Figure 1 from the manuscript. However, the figure is still available in the GitHub repository.*

**- Fig 3 can be described directly in the text. Authors should explain how they handle missing data.**

*We excluded Figure 3 and added to the paragraph:*

*"The dataset does not have duplicate data, and only 3 columns contain missing data: patient age column (33.47%), diabetes duration (88.26%), and insulin usage (89.46%). Notably, the latter two are exclusively recorded for diabetic patients."*

**- There are no comments or description for Figures and Tables. If a figure or table is inserted in the paper, it should be presented and commented on and conclusions drawn, when applicable.**

*We thank the reviewer for the comment and added comments on the new discussion section.*

**- A figure describing the proposed framework would help understanding the paper’s goal.**

**- “Dino V2 Base [18]” should be reference [20]**

*We updated reference, since we are not longer using DinoV2, but a convnext approach to provide better results, DinoV2 is not longer used and cited in the paper.*

**- “as can be seen in Table 3 [18,19].” Reference should be updated accordingly.**

*We thank the reviewer for the comment and updated reference in Table 3.*

**Reviewer #2: I have two major concerns:**

**1) Ethical: The authors should not waive themselves for not having the consent from the subjects from whom the retina picture were used. The fact that the pictures were anonymised is not suffisent. The waiver should be given by an Independant Review Commity.**

*We agree with the reviewer and also worry about privacy risks. Prior to the dataset publication, we reviewed the requirements for sharing retinal images and did an expert consultation. As a result, we concluded that retinal scans are biomarkers, however, since there is no matching dataset, the reidentification risk is low. In our dataset, we only share the data under credentialed users and after human data handling training, and the data sharing will be interrupted in case the reidentification risk increases.*

**2) Adjudication of the diagnostic and other data: it is not sufficient that a single expert review all the pictures. It should be at least 2 independant experts with a mechanism for resolving disagrements. This would add significantly on the credibility of the data.**

*We agree with the reviewer that a single expert review may be limited for labeling, and we added this as a limitation and for future improvement.*

*New paragraph:*

*A single expert evaluation risks introducing bias and undermining the credibility of labels. To enhance the robustness of the BRSET, our future steps target employing two ophthalmologists for labeling, supplemented by adjudication from either a retina specialist or a glaucoma specialist.*

**Reviewer #3: In this work, the authors present the BRSET database, which includes information from retinal photographs of 8,524 Brazilian patients, including sociodemographic and clinical information labeled by a retina specialist. This data is available on PhysioNet (although not referenced as required by the site). The authors detail the dataset and the different labeling stages comprehensively. Additionally, the authors conduct classification experiments using classic machine learning techniques such as SVM and LR to diagnose diabetes, classify by sex, or diagnose diabetic retinopathy. The document is clearly written, but I have some observations that, in my opinion, need to be addressed before publication.**

**Major:**

**- I understand that the experiments have been used as a basis; however, it is not clear why these tasks were chosen. I suggest incorporating a proper justification based on the literature of why these tasks are relevant for this database.**

*We added the following paragraphs to the Experiments section:*

*As diabetic retinopathy is the leading cause of preventable blindness among adults and the most explored disease in publicly available datasets* [*[13,33,34]*](https://paperpile.com/c/K0oJ1u/poLMI+wtwA+kpy3)*, we included diabetic retinopathy prediction and clinical diagnosis of diabetes mellitus through retina fundus photos.*

*To assess the ability to predict patient demographic characteristics through retinal fundus photos, extending beyond pathological findings, we included the prediction of patient sex, which is reported in previous studies* [*[35,36]*](https://paperpile.com/c/K0oJ1u/ftWn+XgPG)*.*

**- Considering the amount of data I deem sufficient, the results do not seem to be promising. I would ask the authors to provide their insight regarding the results compared with the state of the art. Are these metrics common in other types of datasets? Is there information that this experiment is providing that contributes to the differentiation between classes of the datasets?**

*We improved our methods and results, comparing with previous sate of the art reports.*

Minor:

**- Add the standard deviation of age in the abstract.**

*We added the age standard deviation in the abstract.*

*"... and the average age is 57.6 (± 18.26) years."*

**- Include information about the results of the machine learning techniques in the abstract.**

*A ConvNext V2 model was trained and evaluated on BRSET in fourthree prediction tasks: “binary diabetic retinopathy diagnosis (Normal vs Diabetic Retinopathy)” (AUC: 97, F1: 89); “3 class diabetic retinopathy diagnosis (Normal, Proliferative, Non-Proliferative)” (AUC: 97, F1: 82); “diabetes diagnosis” (AUC: 91, F1: 83); “sex classification” (AUC: 87, F1: 70).*

**- More examples could be added in Figure 2 (or additional figures) that contribute to the differentiation between different classes for various tasks. These visual differences would better contribute to the discussion not only of the article but also of future research**

*We thank the reviewer for the suggestion and added multiple diseases represented in the dataset to the new Figure 2 to illustrate better ophthalmological conditions.*
