# Supplementary material for: BRSET: A Brazilian Multilabel Ophthalmological Dataset of Retina Fundus Photos
Source: PLOS Digit Health. 2024 Jul 11;3(7):e0000454. doi: 10.1371/journal.pdig.0000454 (PMC11239107; doi:10.1371/journal.pdig.0000454)
Supplement: Attachment — Submitted filename: Rebuttal letter R2.docx [file pdig.0000454.s003.docx]

Dear Editor,

Thank you for handling our reviewed article and for providing comments that have helped us improve our work. We have revised the manuscript based on the reviewer's suggestions and provided a point-by-point response below. Therein, the Reviewers’ comments are in bold, and our responses are in italics.

To ease the review process, we append a revised version of the manuscript, in which changes are highlighted in red. We hope that you will find the revised version of our work suitable for publication after this round of review.

**Reviewer #4: Based on the reviews provided to your paper, there are some points that can be amended or clarified:**

**1. Additional examples can be added in Figure 2 or a new format can be added to facilitate differentiation between different categories in multiple tasks.**

*We added more examples in Figure 2 and created a new Figure 3 to ease the differentiation between different categories.*

**2. More details about the selection of proposed tasks and experiments can be clarified and the feasibility of these choices can be emphasized based on scientific literature.**

*Diabetic retinopathy was selected as an important cause of blindness and the most explored disease in ophthalmological datasets.*

*"As diabetic retinopathy is the leading cause of preventable blindness among adults and the most explored disease in publicly available datasets* [*[13,33,34]*](https://paperpile.com/c/K0oJ1u/poLMI+wtwA+kpy3)*, we included diabetic retinopathy prediction and clinical diagnosis of diabetes mellitus through retina fundus photos."*

*Patient sex was a demographic characteristic prediction using retinal fundus photos that was explored in previous studies.*

*"To assess the ability to predict patient demographic characteristics through retinal fundus photos, extending beyond pathological findings, we included the prediction of patient sex, which is reported in previous studies* [*[35,36]*](https://paperpile.com/c/K0oJ1u/ftWn+XgPG)*. "*

*In Table 5, we compared the Resnet architecture (state-of-the-art model) to the ConvNextV2 algorithms results.*

**3. Results from machine learning techniques can be improved and explained and compared to normal performance in other types of data sets.**

*Thank you for your comment and for the opportunity to clarify the main goal and scope of our paper. The primary objective of our research is to provide a data descriptor of the Brazilian ophthalmological dataset (BRSET). This dataset is a significant contribution to the field, offering a rich basis for future research and application development.*

*In alignment with this goal, the implementation of machine learning (ML) models in our study is intended as a means of technical validation for the BRSET dataset. We aim to demonstrate the dataset's utility and reliability by showing how it can be used to train ML models and achieve baseline performances. The focus is not on developing or benchmarking novel machine learning techniques per se, but rather on showcasing the dataset's applicability in a practical context.*

**Reviewer #5: Many thanks for the opportunity to review this important manuscript. Addressing bias in the development and use of artificial intelligence is of vital importance.**

**I acknowledge that the previous review’s suggestions and feedback have been addressed within this revised manuscript.**

**I do however have some suggestions for further improvement.**

**1. “concerns for unfair algorithms resulting from non-representative data and biased models cannot be ignored”. The representativeness of the data set is one of a number of biases that can be introduced during the development and use of AI - it would strengthen this paper if the authors acknowledged this and noting whether the authors sought to consider and address other sources of bias. What residual biases might remain in the model?**

*We improved the paragraph to:*

*"While AI represents a breakthrough technology, unfair outcomes can arise from every step of the AI lifecycle, with models developed using non-representative and local data leading to unequal algorithms [9–12]. Among the sources of biases that can be induced in deep learning, those that stand out the most are those introduced by the data, the model, and the decision-making. A way to tackle biases introduced by data, is providing a representative open source dataset at both the sample size, as well as in knowledge through not only clinical variables but also demographic and sociocultural variables [13]."*

*We added to the limitation subsections:*

*"This project sought to address the issue of underrepresentation in ophthalmological data from Brazil and Latin America. However, biases in AI are complex and multifaceted. While improving data representativeness is a crucial step, it's only one aspect among many that needs consideration."*

**2. “datasets that do not adequately represent those who are disproportionately impacted by the disease lead to biased and harmful algorithms”**

**I suggest the authors provide examples to demonstrate this point.**

*We added the paragraph to provide examples:*

*" For instance, conditions like diabetic retinopathy and infectious retinopathies have a disproportionately higher prevalence in patients from LMICs, yet these individuals are often underrepresented in ophthalmological datasets."*

**3."The Brazilian Multilabel Ophthalmological Dataset (BRSET) represents a significant stride towards mitigating biases in healthcare research"**

**The authors do not mention whether the model was evaluated post training to provide assurance that it was performing as intended and adequately addressing bias? If not, this would be a limitation and the authors might want to suggest that further work on this might be required as well as addressing other sources of bias in AI development.**

*We agree, added as a limitation that we haven't evaluated post-training, and stimulate future work on model deployment.*

*"Future work should analyze and address different types of social biases included in our dataset and during model development as well as the use of models, methods such as policy loss function, and model deployment."*

**Reviewer #6: This is a very valuable contribution to the literature. The authors have adequately addressed all of the reviewer comments and have made additional changes in accordance with the reviewer comments. The manuscript is quite improved as a result.**

**An aside, the use and discussion of saliency maps in this context is particularly valuable to the larger discussion on interpretability and explainability and to the larger discussion on equity in the integration of AI in healthcare.**

*We thank the reviewer for the comment.*

**Reviewer #7: This this work, the authors present the BRSET database made publicly available on PhysioNet. This include the information of 8 524 Brazilian patients, including sociodemographic and clinical information labeled by just one retina specialist. The authors addressed most of the comments and suggestions given in the past and the improvements are noticeable, mainly in the performance of the presented models. Also added most of the limitations of this work in the conclusions. The document is clearly written, but there are some sections that do not have the appropriate tone to sound polished and professional. In my opinion, this work needs to address the following points.**

**Major:**

**- As mentioned by Reviewer # 2, there is big ethical concern regarding the data. They provided a comprehensible response to this reviewer, but this is not commented in the main text. Do the authors had the permissions to bring out of Brazil to be published in a server that may be located in the US? What does the Brazilian data laws (if exist) recommend?**

*Brazilian law allows anonymized and, for research purposes, data sharing on external servers. It's worth noting that in our case, retinal images do not consist of identifiable data due to the absence of a matching database, and therefore are not PHI.*

**- I agree with reviewer #2 and being that you may not be able to re-run the labelling with another retina expert, you gave that response that is understandable. However, what about when these 2 clinicians provide different diagnosis/label? How do you decide which is the right label? Maybe this should be considered for future iterations.**

*We agree with the comment and stimulate community collaboration for relabeling in future iterations. We are in the process of relabeling through expert collaboration and will publish the newly revised labels on the Physionet platform and Github repository.*

**- References. Even though this is not a PhD thesis, there are some parameters that should be complied. There are a lot of methods and equations mentioned in the text that do not have the appropriate reference. For instance, cross-entropy loss (line 255, pp 14) where the authors took this equation or method? Where does another student will find this information if they want to apply the same method? This comments applies to all equations (1 - 6). Adam optimizer (pp 15). Softmax (pp 14). Salience maps (pp 17), who said that?**

*We agree with the reviewer and added references to the equations and concepts throughout the manuscript.*

**- Interpretability. For whom is interpretable your findings. This is not clear in the text. As a Data Scientist, it is not completely clear.**

*We added "clinical" and "clinicians" to clarify the role of interpretability saliency maps for clinicians.*

*"To enhance model clinical interpretability, we generated saliency maps for select images across classes"*

*and*

*"Interpretability methods, such as saliency maps, play a pivotal role in demystifying the decision-making process of AI models for clinical practitioners. "*

**Minor:**

**- Why do you add equations that are not referenced in the main text? Precision and recall equations, above equation 5.**

*We added the precision and recall references.*

**- I am afraid that the font style is not appropriate to reference equation variables or other mathematical variables in the main text. Normal font instead of italic?**

*We thank the reviewer for highlighting the mistake. We reviewed all the equations and citations according to the APA style.*

**- Limitations (pp 19). Why don’t you mention (Again) the normal and mild cases? By this time the reader already forgot these values. At least, refer to the table that contains that information.**

*We added the reference for Table 4 in the limitations sections.*

**- There are some contractions that I do not find appropriate in an scientific work in a high profile journal: We’ll …. There’s. Correct them please.**

*We thank the reviewer for highlighting this problem. The new reformulated the paragraphs:*

*"Our primary focus will be harnessing the application of ConvNext V2 as a deep learning method on the BRSET dataset for retina fundus photo analysis"*

*"This implies that if no performance increase is observed over seven consecutive epochs, training ceases, reverting to the last optimal model state."*
